# Using Electron Diffraction and Diffuse Scattering to Investigate the Vacancy Network of Prussian Blue

**DOI:** 10.1063/4.0001030

**Published:** 2025-10-27

**Authors:** Gabriella N. Ruiz, Serhii Vasylevskyi, Michael J. Rose

**Affiliations:** The University of Texas at Austin, Austin, TX, USA

## Abstract

Prussian Blue is considered to be both the first synthetic coordination compound as well as the first modern synthetic pigment. Although it has been used extensively over the last 300 years as a pigment and more recently as an important material for battery applications, a precise description of its crystal structure has been elusive due to the small, disordered nature of it’s crystals and its consequent propensity to be studied by powder x-ray diffraction. Advances in data collection have allowed us to obtain a single crystal structure of Prussian blue using both single- crystal XRD (SCXRD) and microcrystal electron diffraction (MicroED), and to compare the resulting structures, which may provide insight into differences between long-range and local ordering. Additionally, diffuse scattering observed in the MicroED patterns can be reconstructed to reveal relative ordering of vacancy arrangements in the structure that have until now remained hidden from conventional diffraction analyses.

